# Multimodal Ayurveda Intervention in the Management of Recurrent Pilonidal Sinus: A Case Report

**DOI:** 10.1155/cris/1959796

**Published:** 2026-10-18

**Authors:** Wicramanayake W.A.A.P, Peshala K.K.V.S

**Affiliations:** ^1^ Provincial Ayurveda Hospital Pallekele Kundasale Sri Lanka; ^2^ Faculty of Indigenous Medicine University of Colombo Rajagiriya Sri Lanka cmb.ac.lk

**Keywords:** case report, Kshara karma, Nadi Vrana, pilonidal sinus, Shalya Tantra

## Abstract

Pilonidal sinus (PNS) is a chronic inflammatory condition that primarily originates from hair follicles in the natal cleft at the base of the spine and may also occur in other areas such as the axilla and umbilicus. A 25‐year‐old male patient presented to the outpatient department (OPD) of the surgery unit at the National Ayurveda Teaching Hospital, Borella, in November 2022 with complaints of pain and purulent discharge from the sacral region near the anus, accompanied by intermittent fever for 5 days. Clinical examination revealed a tender swelling in the sacral area with a discharging sinus opening and multiple scar marks from previous surgeries. The patient had previously undergone two surgical procedures (wide excision and primary closure) for the same condition. Based on the clinical findings, the case was diagnosed as a recurrent PNS with abscess formation. Management was initiated with incision and drainage of the abscess, followed by application of a semisolid paste of *Apamarga Theekshna Kshara*, a herbomineral alkaline preparation from the whole plant of *Achyranthes aspera* L., burnt conch shells (a source of calcium carbonate) and a paste of the roots of *Plumbago indica* L. (added to improve and to achieve the maximum potency of *Kshara*). Microbiological culture of the pus revealed the presence of beta hemolytic *Streptococcus* (group G) and coliform. Internal medications, including *Pancha tikta* decoction, *Sudarshana Choorna* (powder), *Triphala Guggulu* (tablets), *Amurta Guggulu* (tablets), and *Kaishora Guggulu* (tablets), were administered to manage the infection and fever. During the postoperative period, the wound was cleansed with a polyherbal decoction (*Pancha valkala*) prepared from the aqueous extracts of the barks of *Ficus religiosa*, *Ficus benghalensis*, and *Ficus racemosa*. Wound dressings were performed using the same decoction mixed with *Apamarga Kshara* powder (herbomineral alkaline paste prepared from the whole plant of *Achyranthes aspera* L.and burnt conch shells) (without adding *P. indica* L roots. Hence, it’s in medium potency). Once a healthy wound bed was achieved, dressing was continued with Seethodaka Taila, a medicated oil known for its wound‐healing properties, for 3 months until complete wound healing was achieved. The patient was followed up for 3 years, during which no recurrence was reported. This case suggests that the multimodel ayurveda management approach may be a potential clinical experience that may encourage further study.

## 1. Introduction

Pilonidal sinus (PNS) is a common and often recurrent inflammatory condition caused by hair penetrating the skin (epidermis) [1]. It is characterized by a cavity containing hair and pus that communicates with the skin surface through a granulation‐lined tract in the buttock cleft at the base of the spine. This suppurative condition most commonly occurs between the ages of 15 and 30 years [2]. The estimated incidence of the disease is ~26 cases per 100,000 people, and it occurs about 2.2 times more frequently in men than in women. PNS represents a common problem in primary care because of its tendency for recurrence following surgery and the need for prolonged and repeated wound care [3]. During the Second World War, the condition was frequently observed among jeep drivers, leading to its being referred to as “jeep disease” [4]. The presentation, diagnosis, and management of atypical PNS (occurring in areas other than the natal cleft) differ from those of sacrococcygeal PNS [1]. Approximately 90% of reported cases of umbilical PNS originate from three neighboring Middle Eastern countries: Iran, Iraq, and Turkey [5]. Studies have revealed that recurrence remains a major drawback of the current surgical management. The reported 5‐year recurrence rate is 45.1% in children, 8.7% in young adults aged 18–21 years, and 11.5% in older adults [6]. Additionally, postoperative complications such as wound infection, seroma formation, and hematoma are not uncommon following primary closure and excision of the PNS [7]. PNS remains a significant clinical challenge due to its high recurrence rate and the need for prolonged wound care following surgical treatment. Frequent hospital admissions and repeated surgeries impose a significant financial burden along with a negative impact on social activities in young patients [8]. Based on its clinical presentation, PNS may be correlated with *Nadi Vrana* [9] described in the Sushruta Samhita. Sushruta advocates *Bhedana karma* (incision and drainage) [10],and *Kshara Sutra* (medicated seton smeared with *Kshara* powder) [11] for the management of *Nadi Vrana*. *Kshara* (all the forms of *Kshara*, including *Theekshna Kshara*) facilitates the removal of the unhealthy tissue and subsequent healing of the sinus tract, thereby reducing the likelihood of recurrence. Although many studies have been reported on the management of PNS using *Kshara Sutra* [12, 13], no studies were identified evaluating the management of recurrent PNS complicated by abscess formation. This case report describes the management of recurrent PNS using an ayurveda management regimen.

## 2. Case Presentation

A 25‐year‐old male patient presented with pain and purulent discharge from the lumbosacral region near the anus, accompanied by intermittent fever, for 5 days. On local examination, a 4 cm scar was observed over the natal cleft, which was a remnant of the previous two surgical procedures. He had a history of two unsuccessful surgical procedures, that is, the laying of an opening of the tract followed by primary closure, for PNS performed in 2019 and 2021. A pointed swelling was noted in the midline, suggesting an impending rupture (Figure 1). He was a lorry driver and had abundant body hair, which may have been contributing factors for the development and recurrence of the disease. According to Nixon and Garza [14], the diagnosis of PNS is primarily based on clinical features. Hence, based on the clinical presentation and history of previous surgical procedures, the case was diagnosed as a recurrent PNS with a pus‐filled cavity. He was a nonsmoker, and no other comorbidities were identified (Table 1). Routine investigations were performed within normal limits.

**Figure 1:**
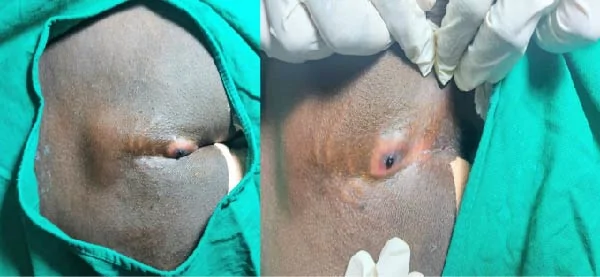
Pilonidal sinus with pus collection inside the cavity and previous surgical scar marks.

**Table 1:** Timeline of the disease.

Year	Event
January 2019	Patient developed PNS and underwent surgery (lay opening of the tract followed by closure of the wound)
May 2021	PNS recurred and lay opening and primary closure was done
November 2022	Symptoms developed
November 6th 2022	Incision and drainage followed by application of *Apamarga Theekshna Kshara* was performed
November 6th 2022 onward	Daily wound dressing
February 2023	Complete wound healing was achieved

During the surgical procedure, the patient was taken to the operating table, and the surgical site was cleaned and draped. After administration of local anesthesia, ~1 cm incision was made over the abscess. Approximately 50 mL of thick, blood‐mixed, blackish‐red color pus was drained and sent for microbiological culture (Figure 2 and Table 2). After drainage, the wound cavity was examined using a metal probe to rule out any communication with the anal canal. On examination, the sinus tract, lined with an unhealthy granulation tissue, was found to extend ~4 cm in the midline toward the presacral fascia. The tract measured ~2 cm in width. Under the guidance of the metal probe, the tract of the sinus was laid open along its entire course. By using instruments, wound curettage and debridement were performed (Figure 3), followed by the application of around half a tea spoon of the thin paste of *Apamarga Theekshna Kshara* over the raw wound area (*Apamarga Theekshna Kshara* is a medicated alkaline paste prepared from the whole plant of *Achyranthes aspera* with increased potency using *Plumbago indica*) (Table 3). *Theekshna Kshara* was washed off with *Pancha valkala* decoction after blackish discoloration appeared over the wound bed (~1 min after application). Complete hemostasis was achieved. The wound was packed with gauze soaked in *Pancha vakala* decoction.

**Figure 2:**
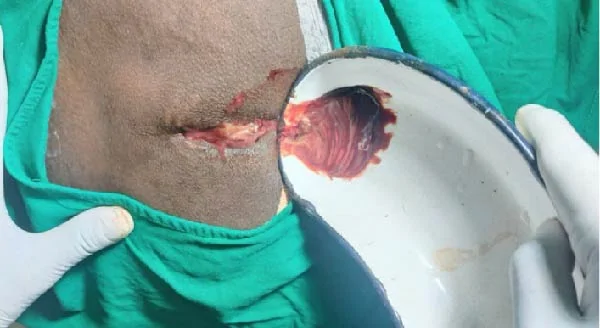
Incision and drainage of the abscess cavity. Blood mixed pus was drained through the incised wound.

**Figure 3:**
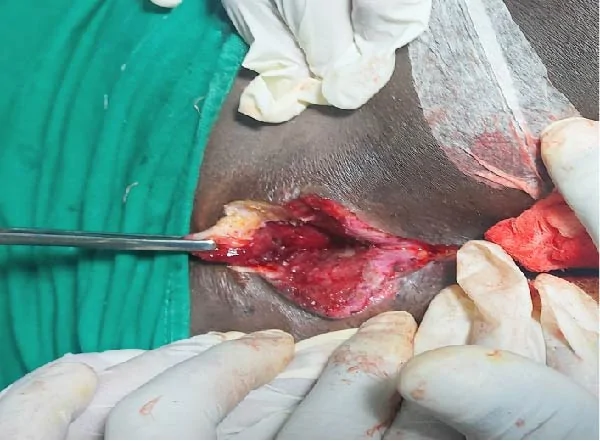
Condition of the wound immediately after debridement following incision and drainage.

**Table 2:** Pus culture and sensitivity report (beta hemolytic *Streptococcus* was revealed).

Culture results	Organism 1 = a coliform isolated
Antibiotic	First line antibiotic
Amoxycillin	Resistant
Cefotaxime	Sensitive
Ceftriaxone	Sensitive
Cefuroxime (oral)	Sensitive
Cefuroxime (parenteral)	Sensitive
Ciprofloxacin	Sensitive
Coamoxyclav	Sensitive
Co‐trimoxazole	Sensitive
Gentamicin	Sensitive
Organism 2 = beta hemolytic *Streptococcus* (group G) isolated	
Antibiotics	First line antibiotic
Cefotaxime	Sensitive
Chloramphenicol	Sensitive
Clindamycin	Sensitive
Erythromycin	Sensitive
Penicillin	Sensitive
Vancomycin	Sensitive

**Table 3:** Details of the ingredients of Kshara.

Apamarga Theekshna Kshara	Apamarga Kshara powder
Ingredients One part of the ash of the whole plant of *Achyranthes aspera* 1/10th parts of the ash of powder of heated lime stones 1/100th of the paste of *Plumbago indica* L. roots Potency: Theekshna (high potency) Topical form of the drug: semisolid paste	Ingredients One part of the ash of the whole plant of *Achyranthes aspera* 1/10th parts of the ash of powder of heated lime stones Potency: *Madhyama* (medium potency) Topical form of the drug: powder

During the postoperative period, daily debridement, followed by wound dressing, was performed. For wound dressing, *Apamarga Kshara* powder (medium potency) dissolved in a decoction prepared from the bark of Ficus species was prepared (around two pinches of *Kshara* powder were added to 10 mL of the decoction). After cleansing the wound with the same freshly prepared Ficus species decoction, sterile gauze soaked in the *Kshara*‐containing decoction was applied to the wound once a day. A clean wound bed was achieved within 3–4 days (Figure 4 and 5). After confirming the features of a clean wound bed, wound dressing was continued with Seethodaka Taila, a medicated oil traditionally used to promote wound healing, for 3 months until complete healing of the wound was achieved (Figure 6). Complete wound healing was achieved within 3 months. *Sudarshana* powder, 1 tablespoon twice a day, *Triphala Guggulu*, 1 tablet twice a day, *Kaishora Guggulu*, 1 tablet twice a day, *Amurta Guggulu*, 1 tablet twice a day, and *Pancha tikta* decoction 120 mL twice a day were given as internal medicines for 14 days (Table 4)

**Figure 4:**
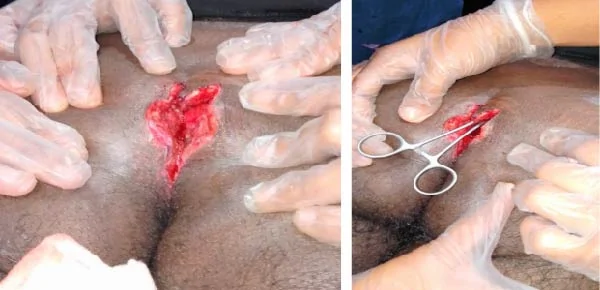
Clean wound 4 days after surgery. The depth and extent of the wound were also reduced.

**Figure 5:**
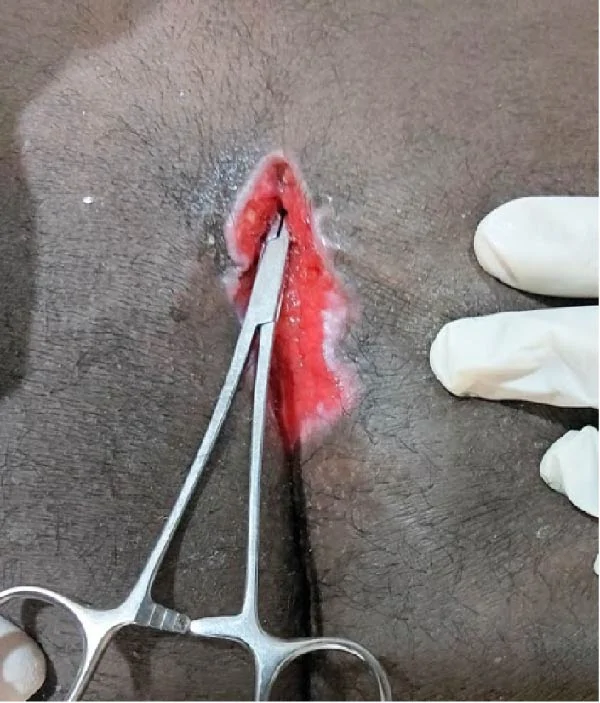
Wound after 1 month of treatment. Instrument demonstrates a reduction in the size of the cavity.

**Figure 6:**
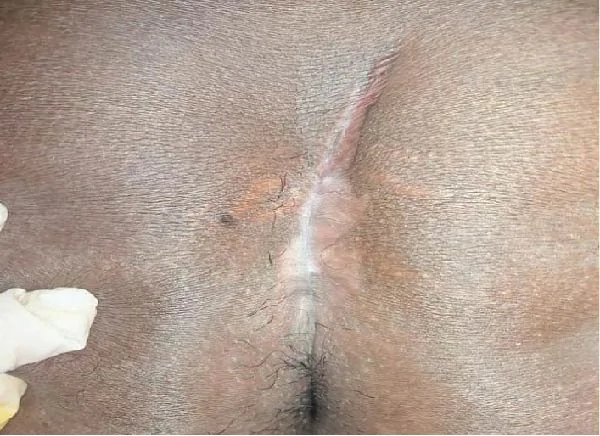
Follow‐up image of the wound 4 months after surgery (1 months after complete healing of the wound).

**Table 4:** Treatment prescribed during the treatment.

Name of the drug	Composition	Dosage	Action
Internal medicines			
Pancha tikta decoction	Nimba (*Azadirachta indica*) Guduchi (*Tinospora cordifolia*) Vasa (*Adhatoda vasica*) Patola (*Trichosanthes dioica*) Kantakari (*Solanum xanthocarpum*)	120 mL twice daily (morning evening) for 14 days	Krimighna (antimicrobial) Purification of blood
Sudarshana powder	Kiratatikta (*Swertia chirata*) Haritaki (*Terminalia chebula*) Bibhitaki (*Terminalia bellirica*) Amalaki (*Phyllanthus emblica*) Black pepper (*Piper nigrum*) Shunti (*Zingiber officinale*) Pippali (*Piper longum*) and other 46 ingredients	1 teaspoonful with lukewarm water every 8 h for 14 days	Antipyretic Antimicrobial Antioxidant
Triphala Guggulu	Haritaki (*Terminalia chebula*) Bibhitaki (*Terminalia bellirica*) Amalaki (*Phyllanthus emblica*) Black pepper (*Piper nigrum*) Guggulu resin (*Commiphora mukul*)	1 tablet twice daily with lukewarm water for 14 days	Krimighna Purification of blood
Amurta Guggulu	Amurta (*Tinospora cordifolia*) Guggulu resin (*Commiphora mukul*) Haritaki (*Terminalia chebula*) Bibhitaki (*Terminalia bellirica*) Amalaki (*Phyllanthus emblica*) Black pepper (*Piper nigrum*) and several other herbs	1 tablet twice daily with lukewarm water for 14 days	Krimighna Purification of blood
Tablet Kaishora Guggulu	Guduchi (*Tinospora cordifolia*) Amalaki (*Emblica officinalis*) Bibhitaki (*Terminalia bellirica*) Haritaki (*Terminalia chebula*) and few other herbs	1 tablet twice daily with lukewarm water for 14 days	Krimighna Purification of blood
External treatment			
Pacha valkala decoction	*Ficus religiosa*, *Ficus benghalensis*, and *Ficus racemosa*	Used for wound dressing	Wound cleansing and healing Antimicrobial
Seetodaka Taila	Coconut oil (*Cocos nucifera*, *Asparagus racemosus*, *Curcuma longa*, *Azadirachta indica*	Used for wound dressing	Wound healing

The patient reported pain during the application of *Kshara* dressings (VAS score 8); however, the discomfort subsided within approximately 10 min. His tolerance for the procedure progressively increased over time. No postoperative bleeding, delayed healing, or infection was observed during this period.

After a 3‐year follow‐up, no evidence of disease recurrence was identified. The patient’s recurrence‐free status was confirmed through structured telephone follow‐up. Additionally, no treatment‐related adverse effects were reported during or after the ayurveda intervention. The patient was advised to maintain lifelong local hygiene and hair removal in the affected area.

## 3. Discussion

*Apamarga Kshara* is an alkaline medicinal preparation that has demonstrated antimicrobial, debridement, and wound‐healing properties [15, 16]. The pus culture results supported the use of *Kshara* during postoperative care as *Kshara* is known for its *Krimighna* action (antimicrobial action). The activity of *Apamarga Kshara* against *E. coli*, one of the organisms isolated from the pus culture in the present case, has also been reported in another study [17]. Incision and drainage facilitated for pus drainage and appropriate debridement of the tract. Considering the high recurrence rates reported even after wide local excision, both *Apamarga Theekshna Kshara* and *Kshara* powder may serve as effective agent to achieve a clean wound bed, prevent infection, and promote wound healing without recurrence when used in combination with other local medications. A study revealed that *Apamarga Kshara* debrides the sinus tract and liquidates the thick pus [18], which supports the observations of the present case. Published evidence on the ayurveda management of PNS is largely limited to *Kshara Sutra* (a medicated thread coated with *Kshara*) therapy, with very few reports investigating the therapeutic efficacy of direct *Kshara* application [19]. Previous studies have demonstrated the efficacy of *Kshara Sutra* in the treatment of PNS, attributable to its action of mechanical and chemical debridement, coupled with progressive healing of the tract [20]. Regarding the time required for complete wound healing, complete healing was achieved within 3 months in the present case, whereas another case report describing a 7 cm sinus tract treated with *Kshara Sutra* reported complete healing within one and a half months [21]. Although the sinus tract in the present case was shorter, the greater depth and width of the tract, together with its recurrent nature, may have contributed to the longer healing period.

As this case involved a multimodal treatment protocol, the observed clinical outcome may have resulted from the sequential and complementary effects of *Kshara*, other local therapeutic applications, and systemic treatments rather than being attributable to *Kshara* alone.

From an Ayurvedic perspective, the outcome of the present case can be explained through the principle of *Samprapti Vighatana*, whereby the pathological sequence responsible for disease manifestation is interrupted at multiple levels. PNS can be correlated with *Nadi Vrana*, with the involvement of *Rakta* and *Mamsa Dhatu*, leading to chronic suppuration, formation of sinus tracts, recurrent discharge, inflammation, and delayed healing [9].

*Bhedana Karma* (incision and drainage) helps to eliminate the accumulated pus, reduce the inflammatory burden, and restore drainage. Subsequent application of *Apamarga Kshara* facilitates *Lekhana* (scraping), *Shodhana* (purification), and *Chedana* (chemical excision of unhealthy tissue), while contributing to the removal of devitalized tissue and reduction of microbial contamination (*Krimighna*: reducing microbial contamination) [22], and eliminating the unhealthy lining of the sinus tract. These actions may have disrupted the local pathological environment, responsible for persistent infection and recurrence. Following adequate wound purification, local wound care promoted the Ropana (healing) phase by facilitating healthy granulation tissue formation and progressive wound contraction from the base of the cavity. Healing from the base of the wound without leaving residual dead space is particularly important in recurrent PNS because incomplete obliteration of the cavity may serve as a nidus for reinfection and recurrence. Complete healing from the base of the wound without the formation of a residual dead space, is also important for reducing the risk of recurrence. In this regard, *Kshara* may facilitate by promoting effective wound debridement, granulation, and healing from the depth of the wound. Thus, *Kshara* not only performed chemical debridement but also created favorable conditions for complete tissue regeneration. While local therapy directly addressed the sinus tract, the internal therapeutic regimen was intended to address the systemic pathological process by pacifying the vitiated *Doshas*, purifying *Rakta*, improving *Agni*, reducing inflammation, enhancing host defense mechanisms, and supporting tissue regeneration. *Sudarshana* powder is a known antibacterial and antioxidant drug [23], and *Amurta Guggulu*, *Kaishora Guggulu*, and *Triphala Guggulu* are used to purify blood and to promote wound healing. *Pancha tikta* decoction is a blood purifier and promotes sound wound healing [24]. Based on their actions, the combined systemic and local interventions therefore interrupted the disease process at multiple stages, representing a comprehensive approach to *Samprapti Vighatana* rather than merely treating the local wound. This holistic strategy may explain the sustained healing observed without evidence of recurrence during follow‐up. Previously reported recurrences of surgical management [6] provide evidence for successful management of the disease using ayurveda regimen.

Therefore, the application of *Kshara* along with an internal therapeutic regimen aimed at pacifying the *Doshas* may represent a potential alternative to wide local excision or flap repair in selected cases of PNS. However, as this is a single‐case report, the findings require further validation through well‐designed clinical studies involving larger patient populations to establish the safety and effectiveness of this approach. Internal therapeutic interventions may contribute to blood purification and exert antimicrobial effects that support wound healing, thereby supporting a holistic approach to wound management. Accordingly, this case highlights the need for further rigorously designed clinical research to evaluate the safety, therapeutic potential, and broader applicability of ayurveda‐based interventions in the management of recurrent PNS.

## 4. Limitations

Exact wound measurements at each stage, other than those depicted in the clinical photographs, were not available.

## Author Contributions

Concept and design, data collection, scientific writing and editing: Wicramanayake W.A.A.P and Peshala K.K.V.S.

## Funding

The authors received no financial support for this study.

## Ethics Statement

The case report was prepared in accordance with the ethical principles of the Declaration of Helsinki and the relevant institutional and journal guidelines. Ethical approval was not required for the publication of this individual case report as per the applicable institutional policy.

## Consent

Written informed consent was obtained from the patient for the publication of this case report and accompanying images.

## Conflicts of Interest

The authors declare no conflicts of interest.

## Patient Perspective

The patient’s perspective regarding the treatment experience, changes in pain and discharge, and overall improvement was obtained 2 months after surgery.

## Data Availability

The data that support the findings of this study are available upon request from the corresponding author. The data are not publicly available due to privacy or ethical restrictions.
